# A novel autophagy-related long non-coding RNAs prognostic risk score for clear cell renal cell carcinoma

**DOI:** 10.1186/s12894-022-01148-8

**Published:** 2022-12-10

**Authors:** Fucai Tang, Zhicheng Tang, Zechao Lu, Yueqiao Cai, Yongchang Lai, Yuexue Mai, Zhibiao Li, Zeguang Lu, Jiahao Zhang, Ze Li, Zhaohui He

**Affiliations:** 1 Department of Urology, The Eighth Affiliated Hospital, Sun Yat-Sen University, No. 3025, Shennan Zhong Road, Shenzhen, 518033 China; 2 The Third Affiliated Hospital, Guangzhou Medical University, Guangzhou, 511436 Guangdong China; 3 The First Clinical College of Guangzhou Medical University, Guangzhou, 511436 Guangdong China; 4 The Sixth Clinical College of Guangzhou Medical University, Guangzhou, 511436 Guangdong China; 5 The Second Clinical College of Guangzhou Medical University, Guangzhou, 511436 Guangdong China

**Keywords:** Autophagy, Long non-coding RNA (lncRNA), Clear cell renal cell carcinoma (ccRCC), Risk score, Prognosis

## Abstract

**Background:**

As the main histological subtype of renal cell carcinoma, clear cell renal cell carcinoma (ccRCC) places a heavy burden on health worldwide. Autophagy-related long non-coding RNAs (ARlncRs) have shown tremendous potential as prognostic signatures in several studies, but the relationship between them and ccRCC still has to be demonstrated.

**Methods:**

The RNA-sequencing and clinical characteristics of 483 ccRCC patients were downloaded download from the Cancer Genome Atlas and International Cancer Genome Consortium. ARlncRs were determined by Pearson correlation analysis. Univariate and multivariate Cox regression analyses were applied to establish a risk score model. A nomogram was constructed considering independent prognostic factors. The Harrell concordance index calibration curve and the receiver operating characteristic analysis were utilized to evaluate the nomogram. Furthermore, functional enrichment analysis was used for differentially expressed genes between the two groups of high- and low-risk scores.

**Results:**

A total of 9 SARlncRs were established as a risk score model. The Kaplan–Meier survival curve, principal component analysis, and subgroup analysis showed that low overall survival of patients was associated with high-risk scores. Age, M stage, and risk score were identified as independent prognostic factors to establish a nomogram, whose concordance index in the training cohort, internal validation, and external ICGC cohort was 0.793, 0.671, and 0.668 respectively. The area under the curve for 5-year OS prediction in the training cohort, internal validation, and external ICGC cohort was 0.840, 0.706, and 0.708, respectively. GO analysis and KEGG analysis of DEGs demonstrated that immune- and inflammatory-related pathways are likely to be critically involved in the progress of ccRCC.

**Conclusions:**

We established and validated a novel ARlncRs prognostic risk model which is valuable as a potential therapeutic target and prognosis indicator for ccRCC. A nomogram including the risk model is a promising clinical tool for outcomes prediction of ccRCC patients and further formulation of individualized strategy.

**Supplementary Information:**

The online version contains supplementary material available at 10.1186/s12894-022-01148-8.

## Introduction

Renal cell cancer (RCC), the third most prevalent urinary tract malignant tumor, caused a crushing physical and mental burden with 431,288 newly diagnosed cases and 179,368 deaths in 2020 worldwide [1]. As the main histological subtypes of renal cell carcinoma, 75–80% of RCC cases are defined as clear cell renal cell carcinoma (ccRCC) [2]. Although a class of innovative therapeutic strategies was utilized, the overall survival (OS) of many patients remains poor due to the concealment and high recurrence rate of ccRCC [3]. Accordingly, the development of preferable clinically applicable methods and appropriate signatures for personalized treatment is urgently needed to improve the prognosis of ccRCC patients [4].

Autophagy is involved in the processes of autophagosome formation and the degeneration by lysosomes for cellular homeostasis [5]. Recently, a series of researchers demonstrated the critical role that autophagy plays in multiple diseases including tumor metabolism, neurodegenerative diseases, disordered immune regulation, and infectious diseases [6–8]. Additionally, the role of autophagy in kidney cancer has also partly been investigated (e.g., melatonin inhibited the progression of ccRCC by initiating autophagy [9, 10]. Thus, determine, the autophagy-related signatures are of great importance for the diagnosis and treatment of ccRCC.

Long non-coding RNAs (lncRNAs) are defined as transcripts with more than 200 nucleotides that cannot be translated into proteins [11]. In recent years the relationship between aberrant expression of lncRNAs and disease progression has been widely investigated, including in cancers [12], cardiovascular diseases [13], and inflammatory diseases [14], which have been neglected for decades. Recently, studies in a growing number of studies have identified that lncRNAs participate in the initiation and progression of carcinomas decisively by activating autophagy. LncRNA GBCDRlnc1, for instance, was linked to the enhancement of autophagy and poor sensitivity of gallbladder cancer cells to antibiotics [15]. Another lncRNA GAS5 promoted autophagy and inhibited the invasion of colorectal cancer cells [16]. Hence, autophagy-related lncRNAs (ARlncRs) may serve as valuable signatures to construct methods for prognostic prediction, which has been validated in breast cancer [17] and bladder cancer [18]. However, few studies have been performed to investigate the relationship between ARlncRs and ccRCC.

Therefore, we determined ARlncRs in ccRCC and constructed the correlated risk scores in the present study. Furthermore, we established and validated a nomogram model considering certified independent prognostic factors, which provides a new tool for outcome prediction in ccRCC patients and further personalized guidelines for a more favorable strategy. Finally, GO and KEGG analyses were performed to investigate the underlying mechanisms of autophagy involved in ccRCC. We present the following article/case following the TRIPOD Guidelines reporting checklist.

## Materials and methods

### Data acquisition and pretreatment

Both the clinical characteristics and corresponding RNA-sequencing data of ccRCC patients were obtained from TCGA (https://portal.gdc.cancer.gov/) and the ICGC portal (https://dcc.icgc.org). Considering death caused by unpredictable factors, the patient samples whose OS was < 30 days were excluded. Additionally, ccRCC samples that lacked complete data were rejected. All data were available in public, therefore informed consent and institutional ethical approval from patients were not needed.

### Screening for ARlncRs

The Human Autophagy Database (HADb: http://www.autophagy.lu/index.html), the first comprehensive human autophagy database [19], was used to identify autophagy-related lncRNAs (ARlncRs). Based on HADb data, 232 autophagy-related genes (ARGs) were extracted, among which 10 duplicate genes were excluded. The expression data of lncRNAs were obtained from TCGA and ICGC, respectively. Then, Pearson correlation coefficients were calculated to investigate the correlation between ARGs and lncRNAs. ARlncRs were identified by the standard of | r |> 0.7 and *p* < 0.05 in TCGA and ICGC, respectively.

### Establishment and validation of the risk score model

Univariate Cox regression analysis and Kaplan–Meier survival curve (KM) were performed to filter Survival -related ARlncRs (SARlncRs) based on package “glmnet” in R software. SARlncRs were subjected to the multivariate Cox regression analysis for the determination of independent prognostic factors and the construction of the autophagy-related risk score model. The risk score was calculated as the sum of the expression levels of lncRNAs weighted by multivariate Cox regression coefficient ( β): Risk score = βgene(1) * expression level of gene(1) + βgene(2) * expression level of gene(2) + … + βgene(n) * expression level of gene(n). The median risk score was regarded as the cut-off point to divide ccRCC patients into high- and low-risk groups. To preliminarily validate the risk score, the Kaplan–Meier survival analysis was performed for comparison of the prognostic difference between the high- and low-risk groups. Principal component analysis (PCA) was utilized to visualize the expression profiles in the high- and low-risk ccRCC groups. A box-plot diagram and subgroup survival analysis was performed to identify the relationship between the risk scores and clinicopathologic characteristics of ccRCC patients. Moreover, we certified the independent prognostic factors using univariate and multivariate Cox regression analyses.

### Construction and validation of the nomogram 

To evaluate prognosis and guide personalized therapy of ccRCC patients, a nomogram was constructed based on clinical characteristics and risk score using the package ‘rms’ in R. Then, the Harrell concordance index (C-index) and calibration curve were performed in the training cohort, internal validation cohort, and external ICGC cohort to estimate the predictive ability of the nomogram for OS. The closer the C-index achieved to 1, the better its discrimination was [20]. The calibration curve indicated consistency of predicted and actual probabilities, for which perfect prediction is supposed to be on the 45-degree line. The time-dependent receiver operating characteristic (ROC) curves were plotted using the package “survivalROC” in R [21] to evaluate the prognostic accuracy for 5-year OS of the nomogram, risk score model, and clinical characteristics.

### Establishment and Functional enrichment of the lncRNA-mRNA co-expression network

To further understand of the correlation between ARlncRs and target mRNAs, a coexpression network of lncRNAs and mRNAs was established, in which the ARlncRs and autophagy mRNAs were identified through Pearson correlation analysis (absolute threshold coefficient value ≥ 0.5). All calculations and visualization were carried out using the Cytoscape software (version 3.7.2, http://www.cytoscape.org). Functional enrichment of the target mRNAs was carried out using the “Metascape” website for calculations and visualization (https://metascape.org) [22].

### Functional enrichment analysis of differentially expressed genes (DEGs)

The DEGs were identified in the high-risk and low-risk groups by the R package “limma”. I | log2FC |≥ 1 and FDR ≤ 0.05 were considered the threshold of DEGs. To further investigate of the mechanism involved in ccRCC occurrence and progression, DEGs were enriched by the KEGG [23–25] pathway and GO analyses consisting of biological processes (BP), molecular functions (MF), and cell components (CC). All statistical analyses were performed using the R software (version 3.6.2). *p* < 0.05 was regarded as statistically significant.

## Results

### Collecting the expression and clinical features of ccRCC patients

**Fig. 1:**
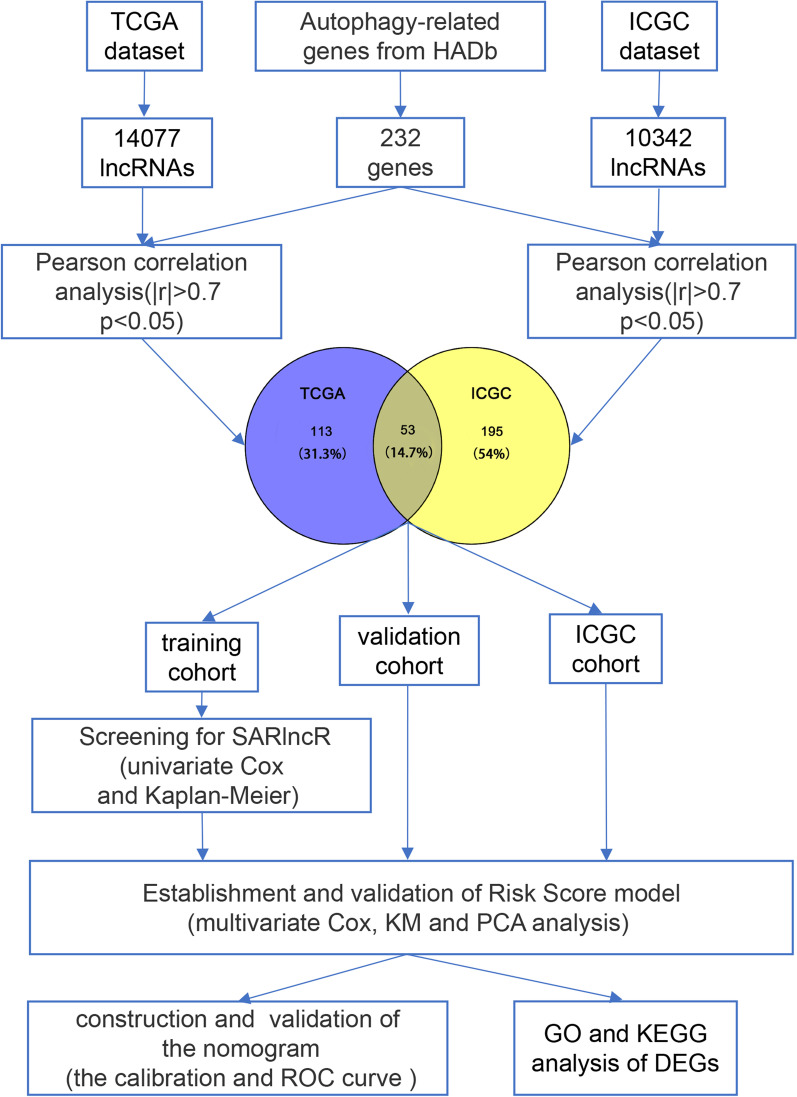
A flow diagram depicted in our study

A flow diagram depicting our study procedure can be found in Fig. 1. The RNA-sequencing and clinicopathologic characteristic data of 483 and 83 ccRCC patients were obtained respectively based on TCGA and ICGC, respectively. The samples in TCGA were randomized into training cohort and validation cohort at a ratio of 1:1. All the cases whose baseline clinical characteristics are presented in Table 1.

**Table 1:** Baseline clinicopathological features of patients with ccRCC in TCGA and ICGC

Variables	TCGA cohort						ICGC cohort (n = 83)	
	Training cohort (n = 242)		Validation cohort (n = 241)		Total (n = 483)			
*Age, n, %*								
< = 65	157	64.88%	162	67.22%	319	66.05%	57	68.67%
> 65	85	35.12%	79	32.78%	164	33.95%	26	31.33%
*Gender, n, %*								
Male	76	31.40%	86	35.68%	162	33.54%	39	46.99%
Female	166	68.60%	155	64.32%	321	66.46%	44	53.01%
*T*								
r	145	59.92%	160	66.39%	305	63.15%	61	73.49%
T3 / T4	97	40.08%	81	33.61%	175	36.23%	22	26.51%
*M*								
M0	202	83.47%	204	84.65%	406	84.06%	75	90.36%
M1	40	16.53%	37	15.35%	77	15.94%	8	9.64%
*Stage, n, %*								
Stage I / Stage II	134	55.37%	154	63.90%	288	59.63%	60	72.29%
Stage III / Stage IV	108	44.63%	87	36.10%	195	40.37%	23	27.71%

### Screening for potential ARlncRs associated with prognosis in ccRCC patient samples

A total of 222 ARGs associated with ccRCC were downloaded from the Human Autophagy database (HADb) analysis. Based on the data of the Ensembl database (http://asia.ensembl.org/index.html) [18], the gene symbols of mRNA and lncRNAs were obtained and annotated from The Pearson correlation coefficients between genes and lncRNAs were calculated to extract ARlncRs. Finally, 168 and 248 lncRNAs were filtered by conducting Pearson correlation analysis using the standard of | r |> 0.7 and *p* < 0.05 in both the TCGA cohort and ICGC cohort (Fig. 1), and a total of 53 lncRNAs overlapped.

### Construction and validation of the risk score model comprising 9 SARlncRs 

Based on the training cohort, 36 lncRNAs were screened by Kaplan‒Meier and univariate Cox regression analyses for prognostic significance in ccRCC. Furthermore, 36 lncRNAs were subjected to multivariate cox regression and 9 SARlncRs were identified for the establishment of the risk score model. These results indicated that SH3BP5-AS1, GARS1-DT, AP000692.1, and AC098484.1 were considered risk factors, whereas the remaining 5 lncRNAs (AC005104.1, CCDC18-AS1, ANKRD10-IT1, AC048382.2, and MHENCR) were considered as protective factors. A risk score comprising 9 selected ARlncRs was constructed with the following formula: Risk Score = 0.451 × expression value of AC005104.1 + 0.131 × expression value of CCDC18-AS1 + 0.101 × expression value of ANKRD10-IT1 + (−0.655) × expression value of SH3BP5-AS1 + (−0.490) × expression value of GARS1-DT + (−0.455) × expression value of AP000692.1 + 0.731 × expression value of AC048382.2 + 0.106 × expression value of MHENCR + (−0.757) × expression value of AC098484.1.

**Fig. 2:**
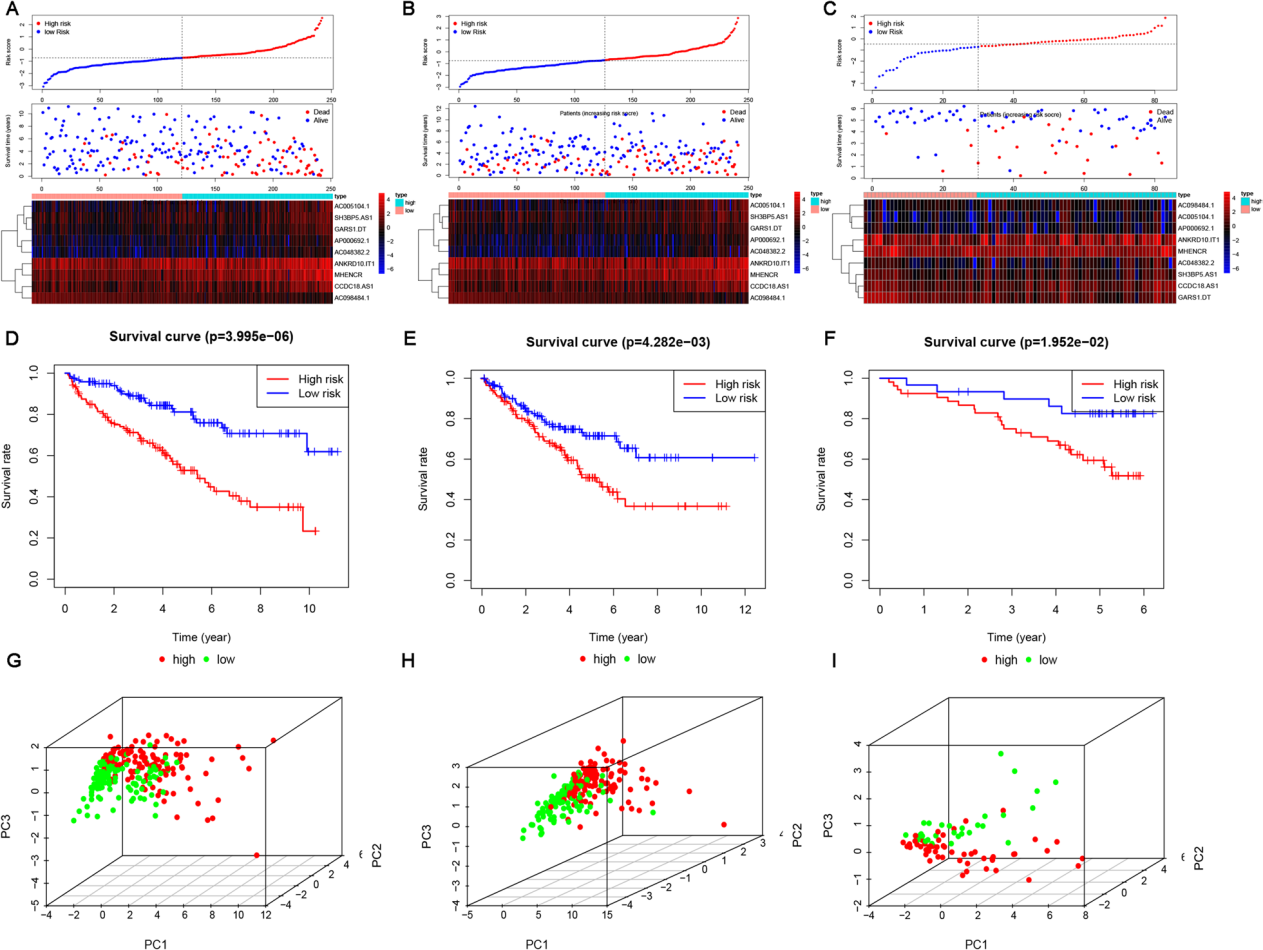
Construction and validation of the risk score model. **A**–**C** Distribution of risk score, scatter plot of survival time, and expression levels of 9 lncRNAs between the high- and low-risk groups in the training cohort, validation cohort, and external validation cohort (ICGC); **D**–**F** Kaplan–Meier survival curve between high- and low-risk groups in the training cohort, validation cohort, and external validation cohort (ICGC); **G**–**I** PCA based on the nine selected ARlncRs between high- and low-risk ccRCC patients in the training cohort, validation cohort, and external validation cohort (ICGC)

Then, ccRCC patients in the training cohort, internal validation cohort, and ICGC cohort were stratified into high- or low-risk groups by calculating risk scores for further estimation of their prognostic evaluation ability. The distribution of risk scores and the scatter plot demonstrated that a higher risk score tended to indicate a worse prognosis of ccRCC patients in the training cohort, validation cohort, and ICGC cohort (Fig. 2A–C). The Kaplan–Meier curve results revealed significant differences (*p* < 0.001) in the prognosis of the two groups. The OS of the high-risk group was significantly poorer than that of the low-risk group not only in the training cohort but also in the validation cohort and ICGC cohort (Fig. 2D–F). In addition, PCA displayed distinct different distribution patterns of high- and low-risk groups (Fig. 2G–I).

### Prognosis and correlation analysis of risk score and clinicopathologic characteristics

**Fig. 3:**
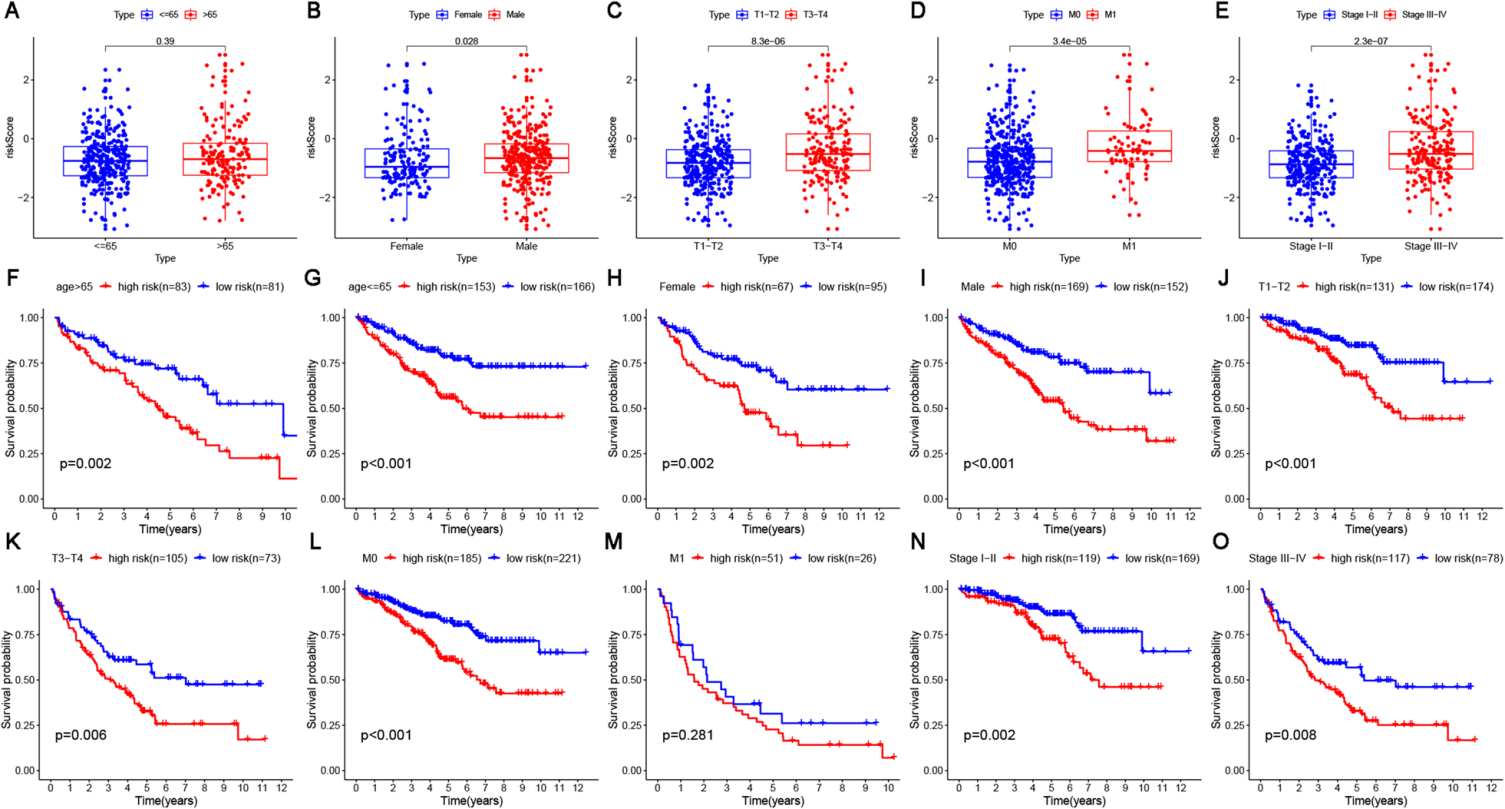
Prognosis and correlation analysis of the risk score with clinical characteristics in TCGA. **A**–**E** Boxplots of the Wilcoxon test of the risk score in clinical characteristics including **A** age **B** gender **C** T stage **D** M stage **E** AJCC stages; **F**–**O** Kaplan‒Meier survival curve between high-risk and low-risk groups ccRCC patients stratified by **F**, **G** age(> 65, <  = 65), **H**, **I** gender (female, male), **J**, **K **T stage(T1–T2, T3–T4), **L**, **M** M stage(M0, M1), **N**, **O** tumor stages (stage I–II, stage III–IV)

Box-plot diagrams delineated about the risk score and clinicopathologic characteristics of the ccRCC patients from TCGA (Fig. 3A–E), and the *p*-value obtained by the Wilcoxon test revealed that male (Fig. 3B), T3-T4 (Fig. 3C), M1 (Fig. 3D), and stages III–IV (Fig. 3E) were significantly associated with the risk score. Furthermore, the prognostic ability of the risk score was assessed by the stratification analysis, whose results (Fig. 3F–O) illustrated that the OS of the high-risk groups was significantly lower that of the low-risk group, whether grouped according to age(> 65, <  = 65), gender (female, male), T stage(T1-T2, T3-T4), M0 stage or stage (stage I-II, stage III-IV). However, no evidence was found for statistically significant differences from the OS between high-risk and low-risk scores in the M1 group, probably attributed to its limited samples (high risk, n = 52; low-risk, n = 26). In short, it was confirmed that the risk score might be valuable in predicting ccRCC patient prognosis.

### Determination of independent prognostic factors

**Fig. 4:**
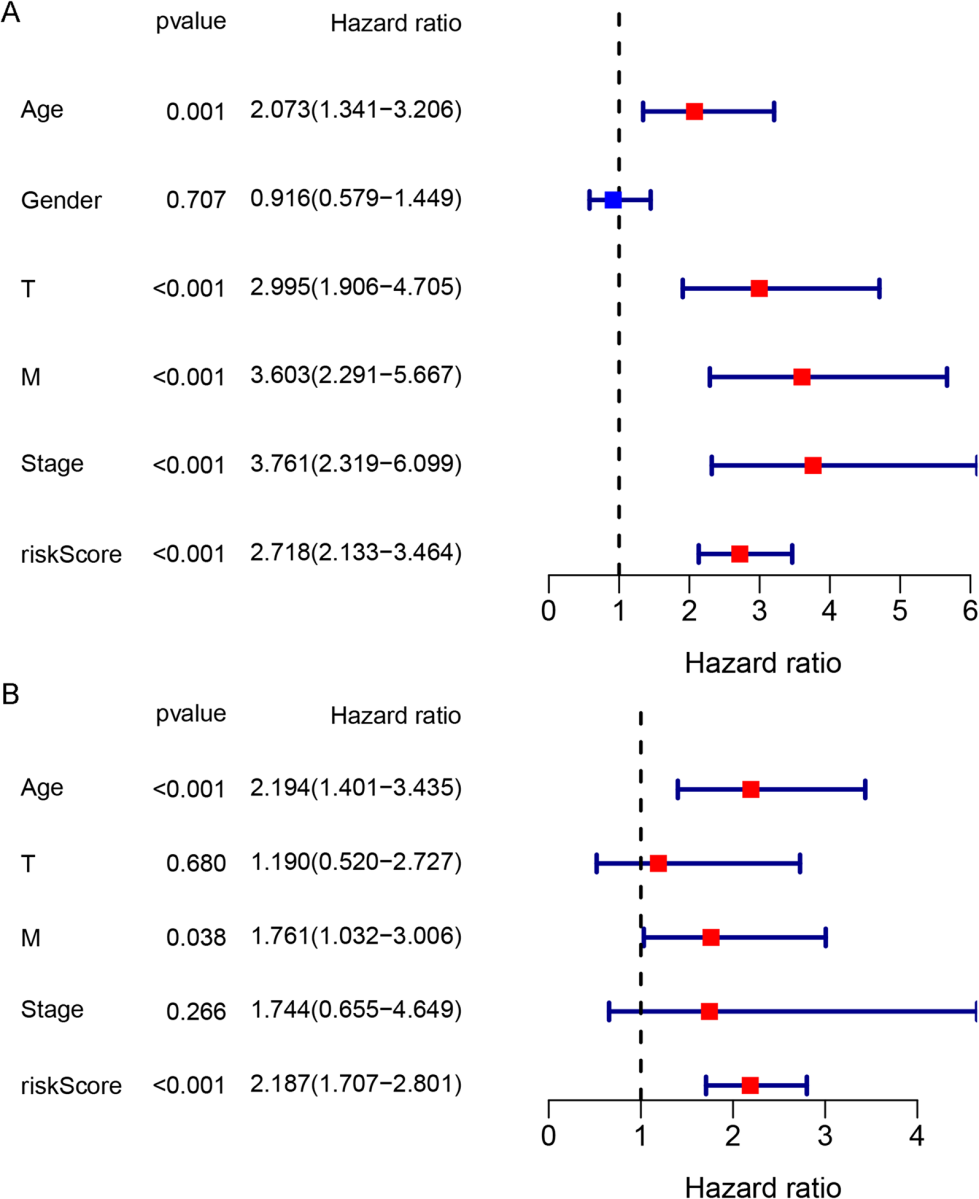
Forest plot of the univariate **A** and multivariate Cox **B** regression analyses of the correlation between the OS of ccRCC patients and clinicopathological features (including risk score) in the TCGA training cohort. The red squares indicate the HR, and the blue transverse line indicates the 95% CI

To further validate the predictive power of the risk score on prognosis and filter potential independent prognostic factors, univariate and multivariate Cox regression analyses were implemented. The results of the univariate analysis indicated that age, T stage, M stage, and risk score were correlated with the OS of ccRCC patients in the TCGA training cohort (Fig. 4A). As shown in Fig. 4B, multivariate Cox regression analysis confirmed that age (*p* < 0.001), M stage (*p* < 0.05) and risk score (*p* < 0.001) impacted significantly on OS. These results suggested that age, M stage, and risk score might serve as independent prognostic factors for ccRCC patients.

### Establishment and assessment of nomogram for Predicting Prognosis comprising risk score 

**Fig. 5:**
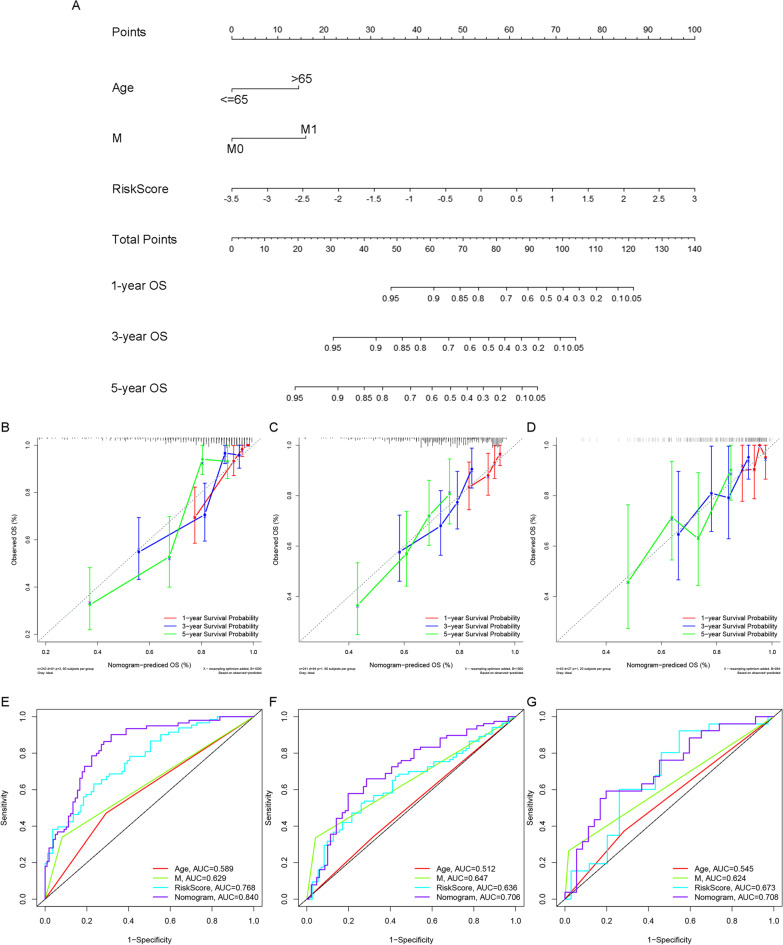
Establishment and assessment of the nomogram. **A** Nomogram considering age, M stage, and risk score; **B**–**D** The calibration curve of the nomogram in the **B** training cohort; **C** validation cohort; **D** external ICGC cohort; **E**–**G** ROC curve of independent prognostic indicators in the **E** training cohort; **F** validation cohort; **G** external ICGC cohort

A nomogram based on defined prognostic factors consisting of age, M stage, and the risk score was established to forecast the 1-, 3- and 5-year survival rates of ccRCC patients as a clinically applicable quantitative scoring method (Fig. 5A). Patients over 65 years with a higher M stage and risk score tend to have a worse prognosis. The C-index of the nomogram using data from the three cohorts was calculated as 0.793(95% CI: 0.744–0.842) in the training cohort, 0.671(95%CI: 0.612–0.730) in the validation cohort, and 0.668(95% CI: 0.567–0.769) in the external ICGC cohort.

Then, the calibration curve certified the favorable consistency between the nomogram and the ideal model-based predictive and practical 1-, 3- and 5-year survival rates in training, validation cohorts, and external ICGC cohort (Fig. 5B–D). Furthermore, the reliability of the nomogram was demonstrated by the ROC curve with the largest AUC in the nomogram. In the training cohort, the result of the ROC curve results demonstrated that the prediction efficiency of nomogram (AUC = 0.840) was significantly superior to that of the other factors including risk score (AUC = 0.766), M stage(AUC = 0.629), age(AUC = 0.589), which was validated in the validation cohort and external ICGC cohort(Fig. 5E–G). In short, these results indicated that the accuracy of the nomogram comprising the risk score to forecast the progression and outcomes of ccRCC patients was significantly superior to that of the conventional method.

### Establishment of the lncRNA-mRNA co-expression network and Functional enrichment analysis 

**Fig. 6:**
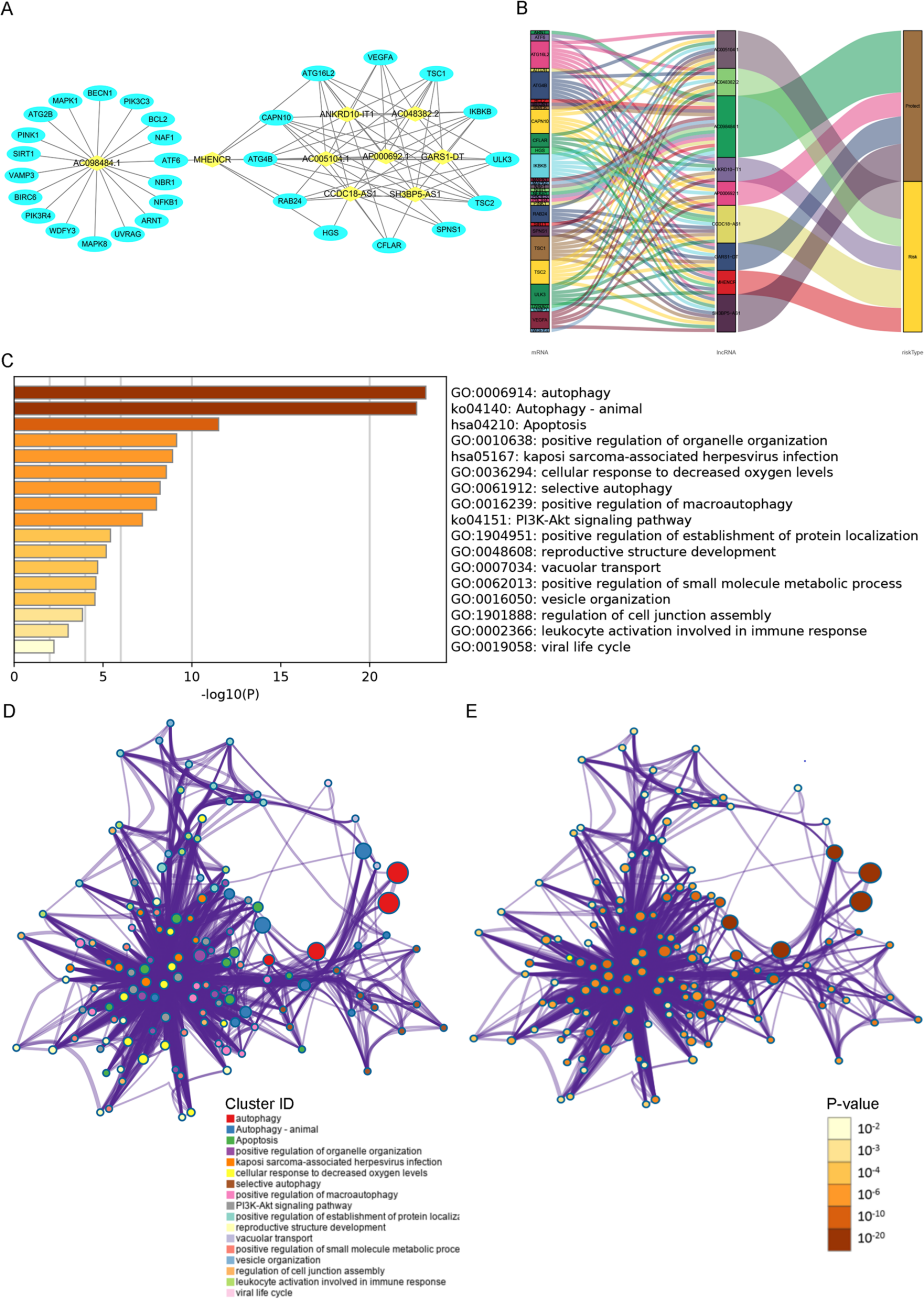
Establishment of the lncRNA‒mRNA coexpression network. **A** The ceRNA network of the 9 SARlncRs and their 30 target mRNAs, whose correlation coefficient was no less than 5. **B** The Sankey diagram of the regulatory relationship between 30 mRNAs, 9 SARlncRs, and risk types (protective or risk); **C** Heatmap of enriched terms, including GO and KEGG, across 30 input mRNAs, colored by p values. **D**, **E** Network of enriched terms for mRNA colored by **D** cluster ID and **E** p value. Nodes closer to each other indicated the same cluster ID. Nodes with more remarkable p valuevalues tended to contain more genes

To further investigate the potential mechanisms of how the 9 SARlncRs are involved in the development of ccRCC, we built a lncRNA‒mRNA network using Cytoscape. Based on preset parameters (correlation coefficient > 5), 30 mRNAs that were highly associated with the 9 lncRNAs were identified, and 88 lncRNA‒mRNA pairs are depicted in Fig. 6A. Next, the regulatory relationship between 30 mRNAs and 9 lncRNAs is shown in the Sankey diagram (Fig. 6B). In addition, we used Metascape to perform functional enrichment analysis of 30 target-related mRNAs, and the results showed that the identified genes mainly function in autophagy, positive regulation of organelle organization, cellular response to decreased oxygen levels, selective autophagy, positive regulation of macroautophagy (GO biological process pathway), apoptosis, Kaposi sarcoma-associated herpesvirus infection (KEGG pathway), PI3-Akt signaling pathway, and autophagy-animal (classical pathway) (Fig. 6C–E).

### Functional enrichment analysis of DEGs

**Fig. 7:**
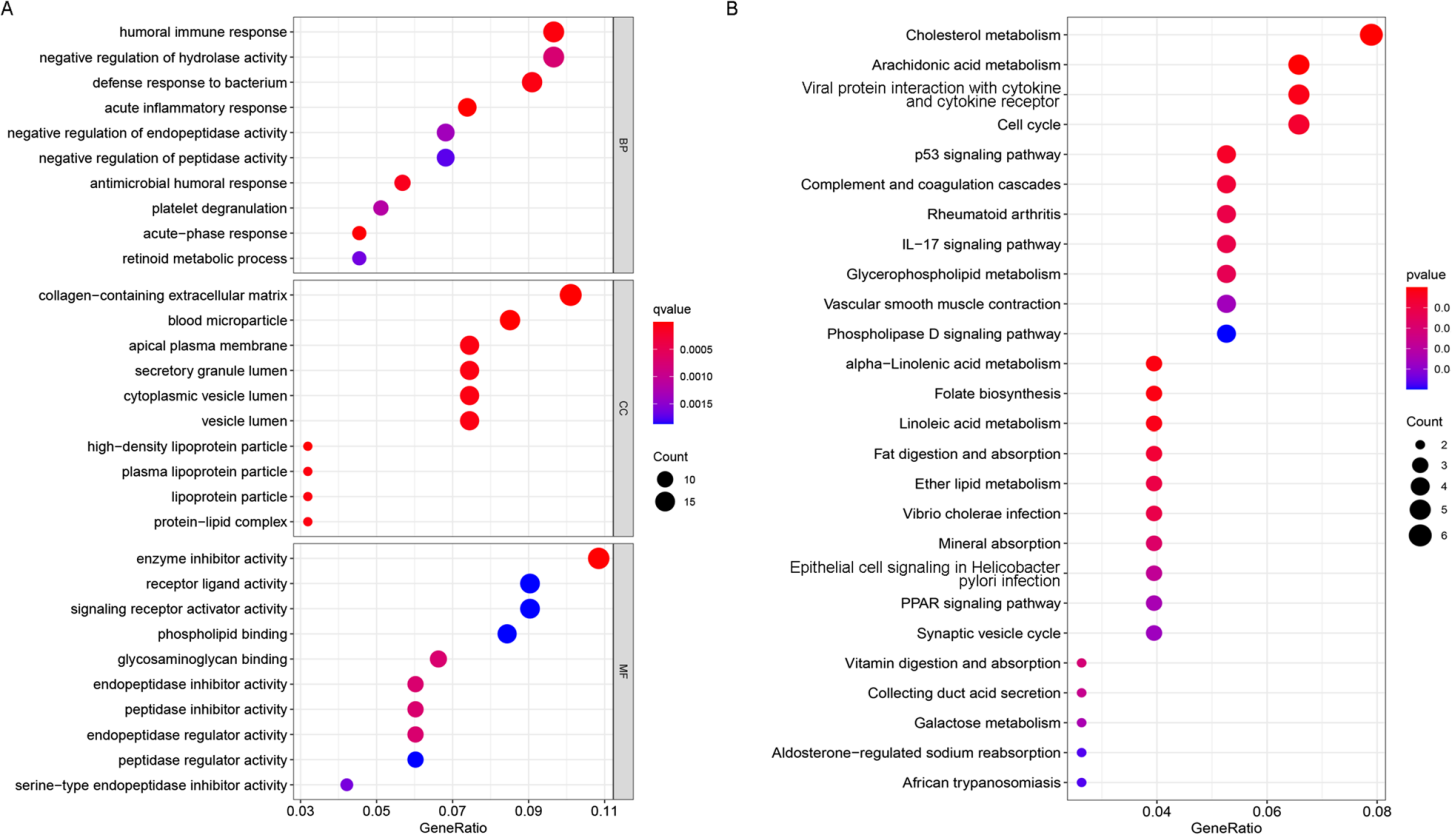
Functional enrichment analysis of DEGs between high- and low-risk groups. **A** Biological processes, cell components and molecular functions in the GO analysis; **B** KEGG signaling pathway analysis with DEGs

Finally, a total of 294 DEGs were discriminated between the high- and low-risk groups (Additional file 1: Table S1). Based on DEGs, GO enrichment analysis and KEGG pathway analysis were applied to investigate the underlying biological mechanisms. As shown in Fig. 7A, acute inflammatory response, acute—phase response, and humoral immune response were enriched in the biological process of GO analysis. Blood microparticles, collagen—containing extracellular matrix, and high—density lipoprotein particles were the top three GO terms for the cellular components. Molecular functions in the GO analysis showed that enzyme inhibitor activity, endopeptidase inhibitor activity, and peptidase inhibitor activity were enriched. Interestingly, KEGG analysis demonstrated that lipid metabolism-related signaling pathways were enriched (Fig. 7B), and the top five enriched terms involved cholesterol metabolism, arachidonic acid metabolism, alpha − linolenic acid metabolism, folate biosynthesis, and viral protein interactions with cytokines and cytokine receptors.

## Discussion

As the main pathological type of RCC, one of the most common urological neoplasms, ccRCC causes increasing health damage year by year. Known for its concealment and insensitivity to chemotherapy, the prognosis of ccRCC patients remains persistently unsatisfactory. Since only subsets of RCC patients respond to a given treatment [26], the identification of predictive biomarkers for treatment selection and sequence is eagerly anticipated. Currently, substantial research has indicated that autophagy could facilitate coping with intracellular and extracellular stress and further affect tumor progression, including several urologic neoplasms, bladder urothelial carcinoma [27], prostate cancer [28], and ccRCC [29]. In addition, mTOR, an autophagy inhibitor, has been approved as a first-line drug that shows bright prospects for autophagy-related therapy for ccRCC patients [19]. Thus, autophagy-related biomarkers may shed light on predicting prognosis and offering guidance for ccRCC therapy. The present studies concentrated on searching for possible applications of ARGs [4, 30], but researchers have been rarely conducted to study the role of autophagy-related lncRNAs in ccRCC.

Recently, an increasing number of researchers have reported the potential of lncRNAs as signatures to predict the occurrence and progression of patients with cancers [21]. Increasing evidence has proven that lncRNAs may be involved in drug resistance and proliferation by regulating autophagy-related pathways in pancreatic cancer, colorectal cancer, and gallbladder cancer [15, 31, 32]. The potential of specific lncRNAs in the proliferation of ccRCC has been widely investigated. Song et al. revealed that the progression of ccRCC could be inhibited by lncRNA ADAMTS9-AS2 via miR-27a-3p-mediated regulation of FOXO1 [33]. Wei et al. indicated that lncRNA URRCC promotes the proliferation of ccRCC [34]. A class of researchers developed a prognostic model considering multiple immune-related lncRNAs [35, 36]; however, the predictive roles of ARlncRs in ccRCC remain unclear.

An increasing number of studies have revealed a significant correlation between ARlncRs and tumor prognosis. Li et al. developed and validated a risk model consisting of 11 ARlncRs that serve as a prognostic prediction tool for breast cancer patients [17]. Wu et al. demonstrated that the dysregulation of lncRNA SNHG11 was correlated with the poor prognosis of patients with gastric cancer by activating oncogenic autophagy [37]. However, existing bioinformatics for ccRCC prognostic models considering lncRNAs are mostly based on a single database and lack a persuasive hypothesis of the mechanism [35, 38, 39]. Past research has identified the correlation between ccRCC and specific genes or immune-related lncRNAs, but the predictive potential of ARlncRs remains to be explored. In our study, a novel ARlncR prognostic risk model was constructed and validated in TCGA and ICGC cohorts.

In the present study, 9 ARlncRs, AC098484.1, AC005104.1, CCDC18-AS1, ANKRD10-IT1, SH3BP5-AS1, GARS1-DT, AP000692.1, AC048382.2, and MHENCR, which have a high correlation with the OS of ccRCC patients, were determined using univariate and multivariate Cox regression analyses. Intriguingly, a risk score model was established to distinguish ccRCC patients into two different risk groups. Then, PCA and ROC curve analysis were utilized to certify the good prognostic prediction ability of the risk score. The subgroup analysis verified that the short OS of patients was correlated with high risk. After that, risk scores, age, and M stage were filtered as independent prognostic factors. Furthermore, we established a nomogram considering independent prognostic factors as a clinically applicable tool to ameliorate the prognosis. Consistent with consensus, patients with high-risk scores and M stage and those over 65 years old are more likely to have poorer outcomes. In addition, as the results of the ROC curve and calibration curve suggested, the nomogram shows high prediction efficiency in not only the training cohorts but also the validation cohorts and ICGC cohorts. In summary, the nomogram including the risk score shows bright prospects in early prognostic evaluation and individualized therapy.

Nine ARlncRs correlated with prognosis were identified and included in the risk score from ccRCC patients. Among these selected lncRNAs, SH3BP5-AS1, GARS1-DT, AP000692.1, and AC098484.1 were considered risk factors, while the remaining 5 lncRNAs, AC005104.1, CCDC18-AS1, ANKRD10-IT1, AC048382.2, and MHENCR, were considered protective factors. ARlncRs were reported to be tightly associated with autophagy and to play an essential role in tumor progression. SH3BP5-AS1 (SH3 domain binding protein 5 antisense RNA1) is affiliated with the lncRNA class. Shao et al. suggested that SH3BP5-AS1 may engage in natural killer cell-mediated cytotoxicity [40], which is directly regulated by autophagy [41]. Related studies have shown that abnormalities in the lncRNA SH3BP5-AS1 are associated with poor prognosis in patients with retinoblastoma by activating carcinogenic autophagy [42]. LncRNA SH3BP5-AS1 can also serve as a necroptosis-associated lncRNA and is included in the necroptosis-associated lncRNA model for predicting breast cancer prognosis [43]. In addition, lncRNA SH3BP5-AS1 is also one of the biomarkers of lung adenocarcinoma and head and neck tumors and is associated with the prognosis of the above two tumors [40, 44]. Published studies of SH3BP5-AS1 are rare, while research on SH3BP5 has been reported frequently. High expression of SH3BP5 was proven to be associated with poor outcomes in acute myeloid leukemia patients [45]. Moreover, SH3BP5 was identified as an invasion- and proliferation-related gene of adrenocortical carcinoma [46]. Therefore, SH3BP5-AS1 may be a signature for predicting the prognosis of ccRCC. GARS1 encodes glycyl-tRNA synthetase, which charges the cognate amino acids of tRNA, and its divergent transcript is called GAS1-DT. Previous studies have indicated that GARS is linked to distal hereditary motor neuropathies and participates in the immunological defense response against the development of tumors [47–49]. These results suggest that GAS1-DT is a potential target for the treatment of ccRCC. In addition, one of the aliases of GARS1-DT is AC005154.6. The MFAP5-miR-200b-3p-AC005154.6 axis as a potential prognostic marker in colorectal cancer may have potential prognostic value in colorectal cancer [50]. CCDC18-AS1 is an antisense RNA1 of CCDC18, a gene that regulates the synthesis of coiled-coil domain-containing protein 18. With the application of exome sequencing, CCDC18 was identified as a candidate susceptibility gene for common familial colorectal cancer [51]. It has been reported that the lncRNA CCDC18-AS1 is not only a regulator biomarker in human breast cancer [52] but also included in the prognostic characteristics of lncRNAs in colon adenocarcinoma [53]. Ankyrin Repeat Domain protein 10 was regulated by the ANKRD10 gene, whose intronic transcript is named ANKRD10-IT1. Six methylation-driven gene biomarkers, including ANKRD10, could serve as a promising predictive model for glioblastoma patients [54]. Furthermore, ANKRD10-IT1 was included in prognostic signatures that may predict the outcomes of patients with hepatocellular carcinoma [55]. These results suggest that ANKRD10-IT1 may act as a signature for the prognostic prediction of ccRCC patients. Melanoma Highly Expressed Noncoding RNA (MHENCR) is a lncRNA that is highly expressed in melanoma. Chen et al. dedicated MHENCR to affecting melanoma progression via the PI3K-Akt pathway [56]. Consistent with our results, PI3K-Akt was regarded as a critical regulator of autophagy. Few studies have been reported on AP000692.1, AC005104.1, AC048382.2, and AC098484.1. According to our analysis, they probably impact the progression of ccRCC via the autophagy-related pathway, but further experimental verification is needed.

Furthermore, genes regulated by the 9 ARlncRs in ccRCC were screened for the establishment of the lncRNA‒mRNA coexpression network. Autophagy-related GO terms and KEGG pathways were enriched in 30 mRNAs correlated with 9 ARlncRs. Network of enriched terms confirming autophagy and apoptosis in ccRCC. Moreover, further bioinformatic analysis of DEGs indicated that immune- and inflammatory-related pathways are likely to be critically involved in the progression of ccRCC, which was validated in existing searches [57, 58]. In addition, according to the results of KEGG analysis, lipid metabolism was determined to be an essential signaling pathway that impacts the prognosis of ccRCC. Wen et al. pointed out that melatonin could inhibit the progression of ccRCC via autophagy and lipid transformation, which were mediated by PGC1A/UCP1 [10]. Zhang et al. investigated how celastrol played a role in ccRCC therapy and found celastrol triggers lipophagy to suppress ccRCC migration [59]. Yuan et al. reported that Mul1 impelled lipid droplets by promoting autophagy and inhibiting the growth and metastasis of tumor cells [60]. Therefore, lipid metabolism and autophagy are complementary to each other in the development and progression of ccRCC.

However, there are also several limitations in the present study that must be noted. First, the accuracy and reliability of risk scores require further validation in more independent cohorts and clinical data. Because clinical samples are from a single database and all belong to the United States, there is a lack of experimental verification, so the results may be biased. Second, since the current research on autophagy-related mechanisms is not completely thorough, the genes in the human autophagy database may not be complete. Last but not least, the nomogram we constructed included 9 lncRNAs, and the number of lncRNAs probably hinders its clinical application. However, it could be feasible in the future with the propagation of high-throughput sequencing.

## Conclusion

In summary, we constructed and evaluated a risk score consisting of 9 autophagy-related lncRNAs, which may improve the accuracy of prognostic prediction of ccRCC. Combined with age gender and risk score, we established a nomogram and proved its reliability as an available method to predict prognosis and guide clinical decision-making. All of these provide a novel insight into the underlying mechanism of ARlncRs on the tumorigenesis and progression of ccRCC.

## Supplementary Information

**Additional file 1**. **Table S1**. The differential expressed genes between high- and low-risk groups

## Data Availability

The mRNA-Seq data and clinical follow-up data associated with the ccRCC patients' samples were downloaded from the TCGA database (https://cancergenome.nih.gov/) and ICGC database (https://icgc.org/).

## Abbreviations

ccRCC: Clear cell renal cell carcinoma
ARlncRs: Autophagy-related long non-coding RNAs
lncRNA: Long non-coding RNA
OS: Overall survival
ARGs: Autophagy-related genes
KM: Kaplan–Meier survival curve
SARlncRs: Survival-related ARlncRs
PCA: Principal component analysis
C-index: Concordance index
ROC: Receiver operating characteristic
DEGs: Differentia expressive genes
BP: Biological processes
MF: Molecular functions
CC: Cell components
HADb: Human Autophagy database
