# Supplementary material for: A novel autophagy-related long non-coding RNAs prognostic risk score for clear cell renal cell carcinoma
Source: BMC Urol. 2022 Dec 10;22:203. doi: 10.1186/s12894-022-01148-8 (PMC9741795; doi:10.1186/s12894-022-01148-8)
Supplement: Supplementary file 1 — Additional file 1. Table S1. The differential expressed genes between high- and low-risk groups [file 12894_2022_1148_MOESM1_ESM.docx]

Supplementary table 1. The differential expressed genes between high- and low-risk groups

| gene | lowMean | highMean | logFC | pValue | fdr |
| --- | --- | --- | --- | --- | --- |
| AC092535.5 | 2.793216693 | 6.418222371 | 1.200246276 | 1.04E-12 | 4.87E-12 |
| AQP9 | 2.906159528 | 5.950006291 | 1.033777295 | 0.012279041 | 0.016803764 |
| AC103702.2 | 1.398341689 | 2.802883786 | 1.003194997 | 1.53E-08 | 4.30E-08 |
| PABPC1L | 2.479434483 | 6.056894331 | 1.288567138 | 1.07E-19 | 1.56E-18 |
| FAM193B | 5.632760228 | 11.30158824 | 1.004611566 | 1.82E-18 | 2.06E-17 |
| IL20RB | 6.333449968 | 15.27299561 | 1.26991957 | 3.93E-11 | 1.51E-10 |
| GADD45G | 3.430925832 | 8.287609956 | 1.272358168 | 5.77E-10 | 1.91E-09 |
| HOTAIRM1 | 2.255397491 | 4.724866989 | 1.066892 | 4.89E-22 | 1.18E-20 |
| C16orf74 | 2.463459801 | 5.553319153 | 1.172664381 | 1.38E-10 | 4.95E-10 |
| AKR1B10 | 4.438354087 | 10.28474017 | 1.212408672 | 2.04E-05 | 3.98E-05 |
| NPR3 | 36.26647743 | 17.21272806 | -1.075160853 | 8.18E-19 | 9.91E-18 |
| AC025265.1 | 0.672052504 | 1.63709866 | 1.284495415 | 4.19E-12 | 1.83E-11 |
| MIR647 | 4.34292264 | 8.818569513 | 1.021878396 | 3.58E-15 | 2.37E-14 |
| LINC02783 | 0.885731745 | 1.914781231 | 1.112237839 | 2.90E-05 | 5.52E-05 |
| TMEM147-AS1 | 0.705507823 | 1.412107753 | 1.001116195 | 1.14E-18 | 1.35E-17 |
| GOLGA8A | 2.336751735 | 4.965557111 | 1.087451125 | 3.78E-12 | 1.65E-11 |
| HSH2D | 0.72522883 | 1.53580565 | 1.082487477 | 5.51E-12 | 2.37E-11 |
| SAA2-SAA4 | 2.292631183 | 6.273231191 | 1.452204446 | 2.09E-07 | 5.13E-07 |
| CXCL1 | 4.937429677 | 13.22956141 | 1.421933128 | 0.005603754 | 0.008036589 |
| NSUN5P1 | 1.22579524 | 2.852966017 | 1.218744555 | 7.38E-25 | 3.63E-23 |
| AL139349.1 | 0.883761373 | 2.375518603 | 1.426513724 | 1.00E-16 | 8.47E-16 |
| RPL13AP25 | 4.245553743 | 8.720156522 | 1.038401295 | 1.36E-05 | 2.69E-05 |
| LINC01554 | 3.304799938 | 7.40916099 | 1.164747244 | 0.017616234 | 0.023582478 |
| INPP5J | 1.064295235 | 4.796316549 | 2.172028469 | 0.010249897 | 0.014208551 |
| MT-TL1 | 2.645457979 | 5.744120014 | 1.118568391 | 1.02E-06 | 2.31E-06 |
| MELTF-AS1 | 0.833503019 | 1.969013255 | 1.240213493 | 1.22E-19 | 1.75E-18 |
| SNORA33 | 2.06700276 | 4.80483236 | 1.216945781 | 1.77E-20 | 3.03E-19 |
| CLIC5 | 2.359255702 | 1.156839002 | -1.028143693 | 2.89E-13 | 1.46E-12 |
| ITIH2 | 0.245262604 | 2.016016188 | 3.039108041 | 2.24E-05 | 4.33E-05 |
| MIR25 | 1.965819185 | 3.935711906 | 1.00149399 | 3.41E-15 | 2.26E-14 |
| SLC10A2 | 7.493064768 | 3.678523469 | -1.026429126 | 2.38E-11 | 9.41E-11 |
| AL354836.1 | 3.103464416 | 6.288193374 | 1.01876598 | 1.26E-15 | 8.99E-15 |
| LINC02604 | 1.643272665 | 3.580427266 | 1.123559877 | 8.51E-25 | 4.12E-23 |
| NFKBIZ | 2.859667015 | 5.832597945 | 1.028291462 | 6.36E-11 | 2.38E-10 |
| YJEFN3 | 0.768043511 | 1.660982859 | 1.112777235 | 1.16E-16 | 9.70E-16 |
| CRYBB3 | 0.832330158 | 2.245435969 | 1.431767766 | 1.66E-05 | 3.26E-05 |
| NBL1 | 7.348864701 | 16.49537029 | 1.166467868 | 2.17E-10 | 7.58E-10 |
| AURKB | 0.896458745 | 2.21276785 | 1.303543004 | 4.42E-16 | 3.38E-15 |
| GTF2IP20 | 1.460782265 | 2.962244046 | 1.019949348 | 3.08E-21 | 6.29E-20 |
| SCG3 | 0.700277555 | 1.510155023 | 1.108697904 | 0.018441205 | 0.024619806 |
| SNORD14A | 2.330233617 | 4.721570295 | 1.01879215 | 1.55E-16 | 1.27E-15 |
| LCN2 | 0.558812524 | 6.502466172 | 3.54055073 | 1.70E-06 | 3.75E-06 |
| AC027796.4 | 0.621335192 | 1.511944942 | 1.282961928 | 2.39E-20 | 3.99E-19 |
| FAM24B | 0.972325275 | 3.643556928 | 1.905836603 | 6.44E-06 | 1.33E-05 |
| DNASE1 | 0.782088853 | 1.710133931 | 1.128704889 | 8.03E-15 | 5.02E-14 |
| AC026403.1 | 3.945363021 | 8.161229539 | 1.048628468 | 4.63E-07 | 1.09E-06 |
| C10orf99 | 9.832973345 | 35.30947267 | 1.844355638 | 0.000229489 | 0.000392526 |
| PPP1R1A | 3.849354765 | 10.25444551 | 1.413560938 | 3.40E-07 | 8.16E-07 |
| AC008610.1 | 0.950954323 | 2.575760014 | 1.437550232 | 9.26E-30 | 2.41E-27 |
| TRIM54 | 1.146701642 | 3.087998963 | 1.4291822 | 0.000939112 | 0.001493868 |
| ITIH3 | 0.621971026 | 2.592884476 | 2.059638648 | 0.003103632 | 0.004607887 |
| STAP1 | 2.511347693 | 8.876938072 | 1.821600351 | 0.018024769 | 0.024088075 |
| HHATL | 1.003789028 | 3.09836812 | 1.626052481 | 0.009161424 | 0.012793222 |
| HABP2 | 26.19135187 | 12.77109593 | -1.036208194 | 0.005072525 | 0.007325387 |
| LOXL1 | 2.590558574 | 5.215350151 | 1.009500912 | 8.06E-08 | 2.08E-07 |
| SERPINF1 | 13.07998361 | 26.36063487 | 1.011024383 | 2.31E-06 | 5.02E-06 |
| GTF2IP13 | 1.024901786 | 2.14843027 | 1.067797287 | 1.84E-26 | 1.39E-24 |
| SLC18A3 | 0.37981636 | 2.082821249 | 2.455165076 | 0.022270411 | 0.029362785 |
| SNTG2-AS1 | 0.410110478 | 2.015389389 | 2.296974097 | 1.01E-12 | 4.74E-12 |
| MTRNR2L8 | 0.714686698 | 1.680793344 | 1.233759512 | 5.04E-11 | 1.91E-10 |
| APCDD1L | 0.640440181 | 2.046003009 | 1.675672536 | 0.031926763 | 0.041162117 |
| AC116914.2 | 0.874998324 | 1.906642817 | 1.123682441 | 7.61E-17 | 6.58E-16 |
| SCNN1D | 0.693199719 | 1.482485404 | 1.096674926 | 1.24E-16 | 1.03E-15 |
| SLC38A5 | 1.927940999 | 6.114636825 | 1.665205912 | 4.18E-05 | 7.81E-05 |
| GABRB3 | 1.850340669 | 0.890178818 | -1.055623835 | 1.55E-16 | 1.27E-15 |
| AL161669.3 | 1.390315064 | 2.793192872 | 1.006503342 | 3.72E-12 | 1.63E-11 |
| SNORD99 | 2.873139921 | 8.980028623 | 1.64409179 | 6.31E-23 | 1.87E-21 |
| FGA | 17.60229311 | 40.33389759 | 1.196229439 | 0.001085282 | 0.001709584 |
| SPTBN2 | 0.932781991 | 2.0026304 | 1.102284347 | 4.75E-08 | 1.26E-07 |
| CHRDL2 | 0.640469123 | 1.53668416 | 1.262619749 | 3.89E-06 | 8.23E-06 |
| MTCO1P53 | 0.360861836 | 2.380799581 | 2.721927698 | 3.41E-12 | 1.50E-11 |
| NPIPB15 | 0.725861027 | 1.763926771 | 1.281025407 | 2.73E-08 | 7.45E-08 |
| AC023669.2 | 2.38434187 | 1.146211512 | -1.056717815 | 0.001618391 | 0.002495168 |
| AVPR1B | 2.465408223 | 1.179567586 | -1.063568465 | 2.73E-14 | 1.58E-13 |
| C19orf33 | 29.80872568 | 68.21876048 | 1.19443384 | 7.45E-09 | 2.17E-08 |
| AL590560.3 | 0.9812597 | 2.510389112 | 1.355204085 | 3.05E-18 | 3.31E-17 |
| ACHE | 0.912952388 | 2.099135859 | 1.201184015 | 1.92E-08 | 5.33E-08 |
| F2 | 1.287666157 | 4.047308342 | 1.652204158 | 1.11E-06 | 2.51E-06 |
| IGKC | 243.1082179 | 609.5777451 | 1.326211571 | 0.007288434 | 0.010303482 |
| RHCG | 8.678848168 | 34.7157821 | 2.000016184 | 1.65E-06 | 3.64E-06 |
| AC021087.4 | 0.995605629 | 2.480769275 | 1.317141272 | 0.003260389 | 0.004826044 |
| PILRB | 0.964479776 | 2.628748421 | 1.446553186 | 3.35E-17 | 3.04E-16 |
| AC006128.1 | 1.052137729 | 2.208297163 | 1.069610753 | 8.18E-17 | 7.03E-16 |
| APOC3 | 0.602118221 | 21.78545556 | 5.177174696 | 0.000546539 | 0.000893155 |
| IGHM | 73.28554343 | 154.6658628 | 1.077554266 | 0.018538955 | 0.024745329 |
| AL161669.2 | 0.991798115 | 2.14611758 | 1.113610731 | 0.002522877 | 0.003788026 |
| CTXN3 | 1.812838898 | 0.82314652 | -1.139029564 | 0.00026093 | 0.000443221 |
| SUCNR1 | 5.491549779 | 2.656890837 | -1.047474398 | 3.56E-11 | 1.38E-10 |
| AGAP6 | 1.126936621 | 2.286584824 | 1.020788058 | 9.36E-20 | 1.38E-18 |
| APOH | 1.30400738 | 18.3339685 | 3.81349516 | 0.000883408 | 0.001409989 |
| SNORD60 | 0.55372029 | 1.469644149 | 1.408237579 | 3.51E-18 | 3.74E-17 |
| CDKN2A | 1.218572367 | 2.649482491 | 1.120518662 | 4.48E-13 | 2.21E-12 |
| MUC12-AS1 | 0.799241362 | 2.215378952 | 1.470850349 | 2.46E-12 | 1.10E-11 |
| SLPI | 22.88434395 | 71.37464144 | 1.64105066 | 2.34E-05 | 4.51E-05 |
| APOA1 | 0.306480871 | 3.573046246 | 3.543285649 | 2.12E-08 | 5.89E-08 |
| SFTPB | 0.61326423 | 2.01608028 | 1.716972377 | 2.54E-12 | 1.14E-11 |
| KLK4 | 1.561693568 | 4.391526041 | 1.491610959 | 4.13E-07 | 9.81E-07 |
| UCN | 0.834024506 | 1.734483257 | 1.056344234 | 3.79E-18 | 4.02E-17 |
| CCN5 | 0.658136243 | 2.420316366 | 1.878737461 | 8.94E-05 | 0.00016069 |
| CAPS | 1.420092812 | 2.896887709 | 1.028518541 | 4.39E-21 | 8.66E-20 |
| IGLC7 | 2.799680589 | 10.60913044 | 1.921972266 | 0.000380476 | 0.00063459 |
| MMP9 | 7.199537719 | 22.32896833 | 1.632940416 | 4.39E-05 | 8.18E-05 |
| ORM2 | 0.172698315 | 6.192920725 | 5.164294068 | 4.05E-10 | 1.37E-09 |
| CRYGS | 0.839912356 | 2.180849951 | 1.376579814 | 1.57E-18 | 1.81E-17 |
| AC023669.1 | 2.198855214 | 1.085265369 | -1.018704758 | 0.000771652 | 0.001239668 |
| TIMP3 | 5.486742462 | 2.498284997 | -1.135011795 | 1.84E-26 | 1.39E-24 |
| MEI4 | 1.888532306 | 0.86122417 | -1.132804749 | 4.78E-12 | 2.07E-11 |
| HERC2P2 | 1.650098196 | 3.397989781 | 1.042129633 | 6.79E-16 | 5.02E-15 |
| PAGE5 | 0.020875681 | 2.286900167 | 6.775426303 | 6.91E-08 | 1.80E-07 |
| PADI3 | 0.483626586 | 1.631721546 | 1.754429423 | 1.37E-05 | 2.72E-05 |
| AC015910.1 | 1.67926779 | 10.45893004 | 2.638831053 | 0.019986372 | 0.026530564 |
| MIR6819 | 0.705737376 | 1.447269493 | 1.036130266 | 2.85E-12 | 1.27E-11 |
| PDCL3P4 | 1.212289115 | 2.513198169 | 1.05179063 | 3.31E-13 | 1.66E-12 |
| SEC31B | 0.691041601 | 1.404596274 | 1.023311043 | 9.30E-16 | 6.74E-15 |
| SLC17A9 | 1.185968334 | 2.945974923 | 1.312679661 | 2.24E-14 | 1.31E-13 |
| MTCO1P12 | 81.66081391 | 187.9808419 | 1.202869784 | 2.54E-15 | 1.71E-14 |
| PYCR1 | 2.226943167 | 4.902296823 | 1.138393099 | 1.61E-07 | 4.00E-07 |
| SPHK1 | 1.237836688 | 2.560483541 | 1.048595297 | 3.37E-10 | 1.15E-09 |
| PLA2G2A | 0.651294834 | 1.55649957 | 1.256922491 | 4.57E-06 | 9.60E-06 |
| LIX1 | 2.123511818 | 1.017615984 | -1.061258902 | 1.98E-05 | 3.85E-05 |
| ITGB2-AS1 | 1.034154592 | 2.27264767 | 1.135922175 | 1.25E-12 | 5.83E-12 |
| NUS1P2 | 4.551969632 | 9.894048939 | 1.120070103 | 0.004252182 | 0.00619631 |
| VSTM2L | 0.511682847 | 2.149141883 | 2.070438953 | 2.37E-10 | 8.24E-10 |
| LRP2 | 47.03570982 | 22.09268774 | -1.090187532 | 9.93E-20 | 1.46E-18 |
| THRSP | 0.741773831 | 1.857456219 | 1.324276928 | 5.85E-06 | 1.21E-05 |
| PLA2G2D | 0.772590419 | 1.700365875 | 1.138069519 | 0.019368009 | 0.025763925 |
| CLNK | 0.751388569 | 2.267343544 | 1.593371928 | 0.000875604 | 0.001397702 |
| MTND4P24 | 0.753330333 | 5.158266312 | 2.775531732 | 6.80E-10 | 2.23E-09 |
| EPS8L3 | 0.865763799 | 2.202897957 | 1.347357285 | 0.001671699 | 0.002570787 |
| MOCOS | 0.841770347 | 1.801669968 | 1.097836168 | 7.83E-08 | 2.03E-07 |
| PPP1R14D | 5.351395913 | 11.10422701 | 1.053121795 | 0.028054602 | 0.036470982 |
| MZB1 | 3.08579179 | 6.642864389 | 1.106164742 | 0.002061709 | 0.003132807 |
| AC020907.4 | 0.715635882 | 1.483888456 | 1.052085017 | 1.09E-13 | 5.86E-13 |
| RNA5SP498 | 0.452860648 | 1.780189206 | 1.974891501 | 4.12E-16 | 3.16E-15 |
| MT2P1 | 0.956574232 | 2.262769789 | 1.24214098 | 4.52E-05 | 8.42E-05 |
| IGLC2 | 137.7177575 | 301.529694 | 1.130585488 | 0.008874583 | 0.01240703 |
| LINC00685 | 0.897183405 | 1.830779818 | 1.028983452 | 4.60E-14 | 2.60E-13 |
| SNORD14E | 1.300681272 | 5.595937728 | 2.10511243 | 1.69E-18 | 1.93E-17 |
| SLC12A8 | 0.944017156 | 2.012052799 | 1.09178318 | 1.21E-07 | 3.05E-07 |
| FER1L4 | 1.613915411 | 3.887543899 | 1.268294001 | 4.90E-16 | 3.71E-15 |
| GPRC5A | 1.86563345 | 3.905555953 | 1.065862368 | 2.05E-06 | 4.49E-06 |
| MT-TC | 8.897134057 | 21.35627842 | 1.263247667 | 0.000378447 | 0.000631602 |
| MTCO1P40 | 8.069807477 | 18.31259002 | 1.182229691 | 4.06E-15 | 2.66E-14 |
| SRPX2 | 2.382092627 | 4.991316629 | 1.067190912 | 0.002111938 | 0.003206194 |
| S100G | 0.126977681 | 3.367029319 | 4.728829447 | 1.17E-05 | 2.34E-05 |
| REG3G | 0.33185945 | 2.108609325 | 2.66764756 | 0.03238606 | 0.041729947 |
| ANGPTL8 | 1.649832926 | 4.483859775 | 1.442421227 | 7.25E-13 | 3.49E-12 |
| SLC25A25-AS1 | 0.874318289 | 1.764548149 | 1.013068315 | 1.09E-14 | 6.69E-14 |
| MTND6P4 | 1.361752919 | 2.751365145 | 1.014682658 | 1.24E-08 | 3.53E-08 |
| SERPINC1 | 0.390542398 | 5.641303716 | 3.852477527 | 0.000129554 | 0.000227989 |
| PGGHG | 15.25467823 | 31.09744001 | 1.027544071 | 4.73E-13 | 2.33E-12 |
| TF | 1.198460515 | 6.675364071 | 2.477664145 | 8.26E-05 | 0.000148947 |
| DUSP15 | 1.010580343 | 2.843436976 | 1.492451804 | 6.74E-05 | 0.00012289 |
| CPNE7 | 0.488705356 | 1.972736642 | 2.013161551 | 3.91E-15 | 2.58E-14 |
| LINC00460 | 0.675858423 | 1.375901223 | 1.02558393 | 0.002218477 | 0.003355652 |
| METTL24 | 1.639653143 | 0.813286158 | -1.01155569 | 4.31E-21 | 8.52E-20 |
| IGHJ3P | 1.525513792 | 4.237824328 | 1.474028561 | 0.001114463 | 0.001752223 |
| SPC24 | 0.89876147 | 1.928710926 | 1.101626747 | 5.68E-20 | 8.71E-19 |
| AL157931.1 | 4.543716443 | 12.64913128 | 1.477093597 | 1.96E-06 | 4.30E-06 |
| SDCBP2 | 1.889376662 | 4.333576348 | 1.197647779 | 0.000728593 | 0.001173761 |
| RDH12 | 1.410086074 | 0.676278349 | -1.060094158 | 0.021306979 | 0.028173693 |
| SYCE1L | 1.110075668 | 2.298947855 | 1.050315722 | 1.13E-14 | 6.92E-14 |
| MFAP2 | 0.862225512 | 2.23331705 | 1.37305092 | 0.000708126 | 0.001142592 |
| GSDMB | 0.817726879 | 1.873347246 | 1.195927377 | 5.38E-21 | 1.04E-19 |
| CPXM1 | 2.069034811 | 4.28540631 | 1.050474078 | 0.001069291 | 0.001685795 |
| PAH | 7.635038709 | 2.790925567 | -1.451891823 | 0.000108109 | 0.000192222 |
| AC015722.2 | 3.763111708 | 7.735317261 | 1.039534348 | 0.038971242 | 0.049684496 |
| C8G | 0.536901107 | 1.784844682 | 1.733070251 | 2.95E-18 | 3.21E-17 |
| CYP4F3 | 0.732157307 | 1.849349956 | 1.336792698 | 0.019082605 | 0.025412202 |
| SAA2 | 3.377662241 | 12.94260235 | 1.938030753 | 4.23E-09 | 1.27E-08 |
| STAG3L5P | 1.95894394 | 3.928506243 | 1.003904741 | 4.16E-14 | 2.37E-13 |
| GOLGA8B | 1.421813706 | 3.434325019 | 1.272294133 | 1.91E-20 | 3.25E-19 |
| IGF2BP2 | 0.879549491 | 2.007185348 | 1.190337181 | 1.59E-05 | 3.13E-05 |
| PI3 | 1.221898132 | 7.662787908 | 2.648745361 | 3.73E-09 | 1.13E-08 |
| CRABP2 | 3.084644588 | 11.42427498 | 1.888926434 | 1.68E-08 | 4.70E-08 |
| BRICD5 | 0.689139105 | 1.479183122 | 1.101933539 | 5.38E-20 | 8.33E-19 |
| SFN | 3.010083227 | 6.823956467 | 1.180805066 | 6.34E-06 | 1.31E-05 |
| UBE2C | 2.956404368 | 9.600837587 | 1.699316663 | 1.33E-18 | 1.55E-17 |
| AC104695.3 | 1.528477639 | 4.477192601 | 1.550498935 | 2.97E-14 | 1.72E-13 |
| AC108673.3 | 1.508761578 | 3.119483048 | 1.047942127 | 1.13E-23 | 3.94E-22 |
| C1QL1 | 15.17607516 | 41.62982979 | 1.455818931 | 2.26E-06 | 4.92E-06 |
| MIR4768 | 3.070648884 | 9.229960807 | 1.587780966 | 7.01E-17 | 6.08E-16 |
| MISP | 0.973838807 | 2.890005605 | 1.569317393 | 1.60E-07 | 3.99E-07 |
| HAGHL | 0.732014809 | 2.026051678 | 1.468726233 | 2.72E-19 | 3.65E-18 |
| INHBE | 0.624623642 | 1.859472992 | 1.573834712 | 7.44E-08 | 1.93E-07 |
| ITPKA | 0.562674809 | 2.47912602 | 2.139458329 | 1.45E-14 | 8.73E-14 |
| PKP3 | 0.650097881 | 1.85372706 | 1.511699983 | 3.44E-10 | 1.17E-09 |
| AC133644.1 | 1.098443707 | 2.249116767 | 1.033897627 | 8.20E-08 | 2.12E-07 |
| ATP6V1B1 | 2.648424398 | 12.02778075 | 2.183164243 | 2.33E-07 | 5.69E-07 |
| AL117379.1 | 0.633425966 | 1.432571609 | 1.177359343 | 1.36E-22 | 3.73E-21 |
| APOA2 | 0.87208947 | 3.00232473 | 1.783531969 | 0.001229225 | 0.001921731 |
| AP000763.2 | 10.53284643 | 21.83213443 | 1.051557816 | 2.98E-09 | 9.10E-09 |
| TNNT1 | 0.366790541 | 2.275637393 | 2.633242351 | 1.24E-09 | 3.95E-09 |
| SERTM2 | 0.312356676 | 1.893872238 | 2.600072738 | 0.000623957 | 0.001013185 |
| SNORD104 | 4.215719758 | 9.923640452 | 1.235090499 | 2.11E-18 | 2.36E-17 |
| IGHGP | 13.93144004 | 32.99009762 | 1.243688656 | 0.025097951 | 0.032859071 |
| AC110285.2 | 1.14091526 | 2.319078421 | 1.023359964 | 2.22E-11 | 8.81E-11 |
| P3H3 | 2.108606188 | 4.49981092 | 1.093574705 | 0.000352124 | 0.000589672 |
| NKD2 | 2.134404411 | 4.680317726 | 1.132772918 | 2.28E-08 | 6.29E-08 |
| SNORD123 | 0.661479053 | 1.395665706 | 1.077186048 | 4.48E-11 | 1.71E-10 |
| MT-TY | 11.40354637 | 26.87940488 | 1.237018641 | 2.55E-05 | 4.88E-05 |
| CDC20 | 2.111310646 | 4.310963815 | 1.029871589 | 2.35E-09 | 7.25E-09 |
| PLK1 | 0.856844195 | 1.719438381 | 1.004832617 | 8.54E-12 | 3.58E-11 |
| CFTR | 1.317418333 | 3.140445793 | 1.253255835 | 8.96E-07 | 2.04E-06 |
| GNRH1 | 0.808029855 | 1.632383623 | 1.014499638 | 1.72E-16 | 1.40E-15 |
| SPINK1 | 2.962816698 | 9.688571655 | 1.709314618 | 1.41E-05 | 2.79E-05 |
| RNU4-62P | 0.833176638 | 3.305018303 | 1.987963969 | 2.54E-13 | 1.30E-12 |
| MTRNR2L12 | 1.890003059 | 4.319619128 | 1.192515542 | 4.42E-13 | 2.18E-12 |
| LINC01089 | 1.280583746 | 2.737063794 | 1.095827457 | 3.62E-23 | 1.14E-21 |
| LMNTD2-AS1 | 2.405527455 | 5.345791685 | 1.152050354 | 6.93E-13 | 3.34E-12 |
| AC027458.1 | 1.921975284 | 0.837993436 | -1.197578935 | 7.16E-15 | 4.51E-14 |
| SNORD100 | 1.189698398 | 2.832710514 | 1.251587294 | 3.11E-14 | 1.80E-13 |
| ASMTL-AS1 | 1.859593824 | 4.673918269 | 1.329644967 | 3.07E-18 | 3.32E-17 |
| AC011462.4 | 1.023391697 | 2.271210499 | 1.15010299 | 1.23E-18 | 1.45E-17 |
| HP | 6.721280177 | 42.31379883 | 2.654320264 | 0.000925019 | 0.001472509 |
| SNHG9 | 2.071978378 | 4.356755767 | 1.072245292 | 3.08E-17 | 2.81E-16 |
| IGHG2 | 123.1429199 | 303.9169036 | 1.303343237 | 0.014976989 | 0.020235095 |
| TNFSF14 | 0.942145799 | 2.217331693 | 1.234802358 | 5.44E-08 | 1.43E-07 |
| EME2 | 1.209799925 | 2.73433838 | 1.176423313 | 1.02E-19 | 1.49E-18 |
| MGAM | 9.444586954 | 4.29107863 | -1.138147366 | 3.59E-18 | 3.82E-17 |
| KLHDC7B-DT | 0.68570416 | 1.497455183 | 1.126854645 | 9.47E-15 | 5.87E-14 |
| GFPT2 | 1.856078491 | 4.424843993 | 1.25336887 | 1.07E-08 | 3.06E-08 |
| RNU4ATAC18P | 1.007427404 | 2.068101488 | 1.037631104 | 8.14E-16 | 5.94E-15 |
| FNDC10 | 0.83760351 | 1.730338595 | 1.046714981 | 1.61E-10 | 5.70E-10 |
| MHENCR | 3.001986173 | 6.624535036 | 1.141901866 | 7.63E-30 | 2.03E-27 |
| TMEM158 | 0.632072394 | 2.2998588 | 1.863383579 | 3.88E-07 | 9.24E-07 |
| CLCNKA | 0.811824606 | 2.854791935 | 1.814145629 | 2.34E-06 | 5.09E-06 |
| BASP1 | 6.628651247 | 14.09104758 | 1.087991615 | 3.71E-05 | 6.98E-05 |
| MTND1P23 | 25.64440059 | 80.41048116 | 1.648739713 | 3.81E-13 | 1.90E-12 |
| MTCO2P2 | 0.639479438 | 1.409513921 | 1.140227851 | 1.41E-16 | 1.17E-15 |
| SLC22A12 | 21.04424404 | 10.37687219 | -1.020054035 | 2.08E-10 | 7.29E-10 |
| CXCL13 | 3.138958003 | 7.380458992 | 1.233424814 | 1.18E-05 | 2.35E-05 |
| AC135050.3 | 0.914416671 | 2.020508173 | 1.143794575 | 4.82E-16 | 3.66E-15 |
| RPL9P29 | 0.673871067 | 1.389340395 | 1.043855619 | 3.78E-20 | 6.08E-19 |
| AC105020.5 | 0.670487412 | 1.425737375 | 1.088426107 | 1.09E-12 | 5.10E-12 |
| NPIPP1 | 1.490478631 | 3.021471122 | 1.019475462 | 1.48E-20 | 2.57E-19 |
| LINC01004 | 1.04432311 | 2.207170076 | 1.079629657 | 6.13E-21 | 1.17E-19 |
| MFSD2A | 0.904169201 | 2.253087215 | 1.31723848 | 3.16E-10 | 1.08E-09 |
| RNU2-11P | 0.883599504 | 1.993795017 | 1.17405258 | 8.42E-13 | 4.00E-12 |
| RHBG | 0.671261195 | 2.62198188 | 1.965711566 | 6.71E-06 | 1.38E-05 |
| AL627309.7 | 0.687992637 | 1.747227461 | 1.344602407 | 4.25E-14 | 2.41E-13 |
| ADAM12 | 1.12677284 | 2.456936078 | 1.12466363 | 0.000165034 | 0.000287165 |
| NPEPL1 | 1.865936983 | 4.44111691 | 1.251022286 | 4.48E-29 | 8.75E-27 |
| AC005046.1 | 1.034094354 | 2.086546557 | 1.012749284 | 2.77E-18 | 3.03E-17 |
| KLK1 | 6.521185371 | 32.25841985 | 2.306469635 | 0.000240428 | 0.000410233 |
| STEAP3 | 6.850534153 | 13.77513296 | 1.007777856 | 2.80E-05 | 5.34E-05 |
| AL021707.6 | 1.135608905 | 2.55445082 | 1.169547094 | 9.08E-20 | 1.34E-18 |
| COX6CP1 | 2.031894718 | 4.070901173 | 1.002522547 | 5.05E-05 | 9.34E-05 |
| AP005233.2 | 2.274728314 | 5.151039199 | 1.179169274 | 1.06E-05 | 2.13E-05 |
| PTGES | 3.053721273 | 6.530576427 | 1.096641951 | 6.72E-10 | 2.21E-09 |
| AC103810.3 | 1.518635089 | 3.086556781 | 1.023223084 | 2.73E-13 | 1.39E-12 |
| AC245884.8 | 0.716942386 | 1.831917524 | 1.35342546 | 4.98E-23 | 1.52E-21 |
| KCNJ1 | 1.82577984 | 0.519807757 | -1.812462741 | 0.034764816 | 0.044626055 |
| AQP6 | 1.425834139 | 15.86528552 | 3.475995412 | 0.012818081 | 0.017499937 |
| IGFBP1 | 13.33669478 | 29.08643799 | 1.124945461 | 2.23E-05 | 4.32E-05 |
| SAA1 | 43.28075593 | 159.5697991 | 1.882390024 | 2.96E-10 | 1.01E-09 |
| CLMP | 0.689802377 | 2.076745518 | 1.590069436 | 0.001973182 | 0.003004479 |
| SLC6A19 | 15.48179309 | 5.720557861 | -1.436344825 | 1.18E-11 | 4.87E-11 |
| PLA2G4F | 0.398104708 | 1.75574515 | 2.14086361 | 0.007310967 | 0.010333134 |
| RNU7-40P | 0.800148382 | 2.407345425 | 1.589103698 | 2.31E-13 | 1.19E-12 |
| AC084117.1 | 1.267373586 | 2.938516676 | 1.213246233 | 2.08E-15 | 1.43E-14 |
| TRIM50 | 0.690978939 | 1.932076799 | 1.483438798 | 0.000246669 | 0.000420017 |
| ORM1 | 0.224196964 | 8.793608243 | 5.293616612 | 0.001146556 | 0.001799911 |
| SFRP1 | 3.682516282 | 1.476516752 | -1.318494179 | 0.000835491 | 0.001336724 |
| RPL37P6 | 0.832404947 | 1.665809202 | 1.000865723 | 1.27E-06 | 2.84E-06 |
| RARRES1 | 4.521814048 | 9.060857449 | 1.002745917 | 0.000257727 | 0.000437948 |
| FOXI1 | 2.960375719 | 12.41429503 | 2.068150143 | 0.003117664 | 0.004627168 |
| IGHG4 | 31.46574286 | 69.02050225 | 1.133242969 | 0.016380462 | 0.022039318 |
| C4orf48 | 1.253326913 | 3.934372157 | 1.650370659 | 1.49E-15 | 1.05E-14 |
| AC048341.2 | 1.333252851 | 3.362423137 | 1.334550875 | 2.78E-21 | 5.74E-20 |
| ATP6V1FNB | 0.909422983 | 2.24447618 | 1.303355416 | 7.10E-14 | 3.91E-13 |
| HJURP | 0.685722376 | 1.451644786 | 1.081991967 | 2.27E-11 | 9.00E-11 |
| SNHG25 | 0.704313492 | 1.671565552 | 1.246910308 | 2.36E-18 | 2.60E-17 |
| TMEM174 | 13.03491544 | 6.055361329 | -1.10609627 | 6.37E-10 | 2.10E-09 |
| ATP6V1C2 | 0.800512883 | 2.924262074 | 1.869076087 | 4.11E-13 | 2.04E-12 |
| HAUS7 | 1.116698862 | 2.419474211 | 1.115453372 | 6.23E-12 | 2.66E-11 |
| AC087741.1 | 0.737766326 | 1.559294425 | 1.079657516 | 6.24E-17 | 5.45E-16 |
| IL6 | 3.282487783 | 6.721722511 | 1.034041344 | 0.004955819 | 0.007168547 |
| MYBL2 | 1.594936035 | 3.783451147 | 1.246204251 | 2.82E-10 | 9.69E-10 |
| AC103691.1 | 0.768626127 | 1.882468697 | 1.292271953 | 9.67E-17 | 8.22E-16 |
| MTRNR2L1 | 11.85028418 | 40.03632485 | 1.75638789 | 1.38E-05 | 2.74E-05 |
| DMRT2 | 1.442904568 | 4.631365231 | 1.682461649 | 0.007391334 | 0.010441164 |
| PPP1R14B-AS1 | 0.742094116 | 1.866051114 | 1.330314431 | 9.54E-18 | 9.42E-17 |
| RHPN1 | 2.621385601 | 5.347325233 | 1.02848784 | 1.10E-22 | 3.10E-21 |
| PVALB | 6.568536204 | 33.02912437 | 2.330094915 | 0.00107683 | 0.001696673 |
| TPSG1 | 1.692892792 | 4.667524731 | 1.463167054 | 0.015721107 | 0.021188694 |
| PDGFRL | 1.162495353 | 2.615632989 | 1.169935175 | 3.32E-06 | 7.09E-06 |
| DBH-AS1 | 0.771863636 | 1.67073229 | 1.114062684 | 7.88E-10 | 2.56E-09 |
| AC093001.1 | 0.431584012 | 1.711850865 | 1.987843694 | 1.68E-05 | 3.30E-05 |
| RNY3P16 | 0.764600054 | 2.094668326 | 1.453944616 | 5.33E-16 | 4.01E-15 |
| C11orf86 | 0.901427776 | 3.147666356 | 1.803998817 | 0.000357789 | 0.000598706 |
| AC104958.2 | 1.18549305 | 2.564371209 | 1.113117911 | 0.00741738 | 0.010475727 |
| TTR | 0.316666249 | 4.486214221 | 3.824463498 | 0.001237329 | 0.001933946 |
| CUBN | 46.3758999 | 22.28143445 | -1.057533162 | 1.61E-18 | 1.85E-17 |
